# Preliminary Molecular Characterization and Antimicrobial Activity of *Enterococcus faecium* Strains Isolated from Algerian Camel Milk

**DOI:** 10.3390/life16071190

**Published:** 2026-07-18

**Authors:** Yasmine Saidi, Mohamed Merzoug, Chaimaà Naila Brakna, Keltoum Bendida, Soheir Bouzidi, Marwa Aireche, Hayet Messaoui, Amaria Ilhem Hammadi, Yasmine Zohra Zater, Hadjer Bouderbala, Svetoslav Dimitrov Todorov, Djamel Saidi

**Affiliations:** 1 Genomics Technology Platform, Higher School of Biological Sciences of Oran, BP 1042 Saim Mohamed, Cité Emir Abdelkader (Ex-INESSMO), Oran 31000, Algeria; yasmine.saidi@gmail.com (Y.S.); chaimaa1012@gmail.com (C.N.B.); keltoumbendida2001@gmail.com (K.B.); bouzidisoheir12@gmail.com (S.B.); marwa.air13@gmail.com (M.A.); mess_hayet@yahoo.fr (H.M.); hammadiamaria267@gmail.com (A.I.H.); yassminezater93@gmail.com (Y.Z.Z.); h.s.bouderbala@outlook.com (H.B.); djamsaidi@gmail.com (D.S.); 2 ProBacLab, Laboratório de Microbiologia de Alimentos, Departamento de Alimentos e Nutrição Experimental, Food Research Center, Faculdade de Ciências Farmacêuticas, Universidade de São Paulo, São Paulo 05508-000, SP, Brazil; 3 Department of General Hygiene, I.M. Sechenov First Moscow State Medical University, Trubetskaya St., Bldg. 8/2, Moscow 119435, Russia

**Keywords:** *Enterococcus faecium*, antimicrobial activity, enterocins, bacteriocins, food-associated bacteria

## Abstract

This study characterized six *Enterococcus faecium* strains isolated from Algerian camel milk to assess their molecular features and antimicrobial potential as food-associated isolates. Identification was performed using phenotypic tests, matrix-assisted laser desorption/ionization time-of-flight mass spectrometry (MALDI-TOF MS), and 16S rRNA gene sequencing. Strain diversity was assessed by repetitive sequence-based polymerase chain reaction (rep-PCR), including (GTG)_5_-PCR, BOX-PCR, and enterobacterial repetitive intergenic consensus PCR (ERIC-PCR). Polymerase chain reaction (PCR) screening for selected virulence-associated and antimicrobial resistance genes showed that the two selected isolates were negative for the targeted markers, indicating a preliminary molecular profile of interest but not allowing definitive safety conclusions without whole-genome sequencing and phenotypic antimicrobial susceptibility testing. Both strains showed antimicrobial activity against Gram-positive and Gram-negative indicators. Strain 9 showed notable inhibition against *Klebsiella pneumoniae* subsp. *pneumoniae* ATCC 13883, with an inhibition zone of 23.4 ± 2.3 mm using native supernatant, which increased after concentration. Trypsin treatment abolished activity, supporting the proteinaceous nature of the inhibitory compounds. The enterocin genes *entA* and *entB* were detected in strains 9 and 13, indicating genetic potential only. Further genomic, safety, and functional investigations are required.

## 1. Introduction

Camel milk is a valuable nutritional resource in arid and semi-arid regions, including Algeria, and harbours a diverse microbiota that may influence the safety, quality, and functional properties of traditional dairy products [1,2]. Its reported health-promoting properties have been associated with bioactive components, antimicrobial proteins, and beneficial microbial populations [3,4]. Among the microbiota associated with camel milk, lactic acid bacteria, particularly *Enterococcus faecium* (*E. faecium*), have attracted considerable attention because of their potential contribution to fermentation processes, flavour development, antimicrobial compound production, and food biopreservation. Some *E. faecium* strains have also been investigated for probiotic-related traits, including tolerance to gastrointestinal stress conditions [5,6,7].

Several technologically relevant lactic acid bacteria have been described in camel milk, including species belonging to *Lactiplantibacillus*, *Limosilactobacillus*, *Lacticaseibacillus*, *Lactococcus*, *Leuconostoc*, *Pediococcus*, and *Weissella*, highlighting the functional diversity of this ecosystem [8,9]. These microorganisms may contribute to fermentation, preservation, and the production of bioactive metabolites such as organic acids, bacteriocins, and antioxidant compounds [10,11].

Molecular investigations have shown that camel milk-derived *E. faecium* strains may produce bacteriocins, including enterocins, with inhibitory activity against foodborne pathogens such as *Listeria monocytogenes* and *Clostridium perfringens* [12,13]. These antimicrobial peptides are of interest as natural preservatives because they may contribute to food safety, shelf-life extension, and the reduction in undesirable microbial populations in fermented dairy products. In addition, recent studies have emphasized the technological and antimicrobial potential of *Enterococcus* spp. within the camel milk microbiota, supporting their possible relevance in food biotechnology [14].

However, the use of *E. faecium* in food-related applications requires careful strain-level evaluation. Although some strains may display useful technological or functional traits, *E. faecium* also includes hospital-associated lineages, including vancomycin-resistant enterococci, that may harbour antimicrobial resistance determinants, virulence-associated genes, and mobile genetic elements. The ability of enterococci to acquire and transfer genetic determinants through horizontal gene transfer represents an important safety concern, particularly when strains are considered for food biotechnology applications [15,16,17]. Therefore, screening for virulence-associated genes and antimicrobial resistance determinants represents an essential preliminary step in the characterization of food-associated *E. faecium* strains before considering any technological use.

The selected gene panel was chosen to provide a preliminary assessment of safety-related and functional traits commonly investigated in *Enterococcus* spp. [12,13,15,16,17]. Virulence-associated targets included genes related to cytolysin production (*cylM* and *cylA*), gelatinase activity (*gelE*), adhesion or aggregation traits (*agg* and *ace*), hyaluronidase activity (*hyl*), and pheromone-related determinants (*cob* and *ccf*) [15,16,17]. Antimicrobial resistance targets included genes associated with vancomycin resistance (*vanA*, *vanB*, and *vanC1*) and macrolide resistance (*ermA* and *ermB*) [15,16,17]. Enterocin structural genes were also screened to evaluate the genetic potential for bacteriocin production [12,13].

Accordingly, the objective of this study was to characterize camel milk-derived *E. faecium* isolates using phenotypic, proteomic, and molecular methods and to evaluate their antimicrobial activity against selected indicator pathogens. For this purpose, six *E. faecium* strains isolated from Algerian camel milk were investigated using MALDI-TOF MS, 16S rRNA gene sequencing, rep-PCR genotyping, PCR-based screening of selected enterocin structural genes, virulence-associated genes and antimicrobial resistance determinants, and in vitro antimicrobial activity testing. This approach aimed to provide a preliminary molecular and functional assessment of camel milk-derived *E. faecium* strains and to identify isolates that warrant further genomic, safety, and functional investigation.

## 2. Materials and Methods

### 2.1. Bacterial Cultures and Phenotypical Identification of the Isolates

Six bacterial isolates previously recovered from four raw camel milk samples collected between 2015 and 2016 in southwestern Algeria, specifically in the regions of Ghardaïa and Béchar, were used in this study. The isolates were coded 9, 10, 11, 12, 13, and 17 for laboratory traceability. These isolates were selected from a previously established collection of lactic acid bacteria isolated and preliminarily characterized as described by Saidi et al. [18]. Briefly, camel milk samples were collected directly from lactating camels, transported to the laboratory at 4 °C in an insulated cooler containing ice packs, and processed immediately upon arrival. Samples were serially diluted in sterile peptone water and plated on MRS and M17 agar under selective growth conditions. Isolated colonies were purified by successive streaking steps before preliminary phenotypic characterization.

The isolates were originally preserved in MRS broth supplemented with 30% (*v*/*v*) glycerol and stored at −20 °C. For experimental work, frozen stocks were reactivated by inoculation into fresh MRS broth at approximately 1% (*v*/*v*) and incubated at 30 °C for 24 h under aerobic conditions. For long-term preservation, stock cultures were maintained in MRS broth containing 30% glycerol and stored at −80 °C.

Preliminary taxonomic identification followed standard morphological and biochemical assays for lactic acid bacteria, as previously described by Saidi et al. [18]. These assays included Gram staining, catalase reaction, gas production from glucose using the Durham tube method, tolerance to NaCl at 4% and 6.5%, growth at alkaline pH 9.6, and growth at 45 °C. Presumptive identification as *Enterococcus* spp. was based on Gram-positive coccoid morphology, catalase-negative reaction, growth at 45 °C, tolerance to 6.5% NaCl, and growth at pH 9.6. Only isolates showing traits compatible with the genus *Enterococcus* were selected for further molecular screening and antimicrobial characterization.

### 2.2. Identification and Genotyping of Isolates

#### 2.2.1. MALDI-TOF MS Identification

MALDI-TOF MS identification was performed at the Genomics Technology Platform of the Higher School of Biological Sciences of Oran (ESSBO, Oran, Algeria) using the MALDI Biotyper Sirius GP System (Bruker Daltonics GmbH, Bremen, Germany). Isolates were first grown on MRS agar at 30 °C for 24 h under aerobic conditions. For each isolate, a single colony was deposited onto an MBT Biotarget 96 plate (Bruker), overlaid with 1 µL of 70% formic acid, and allowed to dry. Then, 1 µL of α-cyano-4-hydroxycinnamic acid (HCCA) matrix solution was added before analysis, following the manufacturer’s recommendations and the procedure described by [19].

Spectra were acquired with FlexControl software (version 3.4, Bruker) and processed using MBT Compass HT software (version 5.1, Bruker). Before analysis, calibration was performed using the Bruker Bacterial Test Standard. Identification scores were interpreted according to the Bruker criteria: values ≥ 2.0 were considered reliable for species-level identification, whereas scores between 1.70 and 1.99 were considered reliable only at the genus level. For each isolate, the best-matching species identification and the corresponding MALDI-TOF MS log(score) value were recorded individually.

#### 2.2.2. DNA Extraction

Genomic DNA was prepared from the selected isolates using the PureLink Genomic DNA Mini Kit (Thermo Scientific, Waltham, MA, USA), according to the manufacturer’s instructions. A lysozyme pretreatment step (10 mg/mL) was included to improve lysis of Gram-positive cells, as previously described [19]. DNA quality was assessed with a ScanDrop spectrophotometer (Analytik Jena, Jena, Germany). The A260/A280 ratios ranged from 1.8 to 2.0, while A260/A230 ratios were above 2.0, indicating satisfactory purity. DNA integrity was checked by agarose gel electrophoresis, and the extracted DNA was stored at –20 °C until use.

#### 2.2.3. 16S rRNA Gene Amplification, Sequencing, and Phylogenetic Analysis

For molecular confirmation, the 16S rRNA gene was amplified using the universal bacterial primers 27F and 1495R (Table 1), following previously described conditions [20,21]. Primers were synthesized by the Genomics Technology Platform of the Higher School of Biological Sciences of Oran (ESSBO, Oran, Algeria). PCR was performed in a final volume of 50 µL using 2× PCR Master Mix (Thermo Scientific, Waltham, MA, USA) in a SimpliAmp thermal cycler (Applied Biosystems, Foster City, CA, USA).

The amplification program included an initial denaturation at 94 °C for 10 min, followed by 35 cycles of 94 °C for 45 s, 54 °C for 45 s, and 72 °C for 90 s. A final extension was carried out at 72 °C for 10 min. The amplified fragments, approximately 1500 bp in size, were separated on a 1% agarose gel prepared in TBE buffer, and fragment size was estimated using a GeneRuler Express DNA Ladder (Thermo Scientific, Waltham, MA, USA). After sequencing, raw reads were quality-trimmed before analysis; consequently, approximately 700 bp of high-quality sequence were retained for BLAST comparison and phylogenetic analysis. Phylogenetic relationships were inferred using the maximum likelihood method with 1000 bootstrap replicates, and *Lactiplantibacillus plantarum* was used as the outgroup.

#### 2.2.4. rep-PCR Genotyping of Isolates

To evaluate genetic diversity and confirm that the isolates represented distinct strains, repetitive sequence-based PCR (rep-PCR) was performed using three primer systems: GTG_5_, BOXA1R, and ERIC [21,36,37,38]. Primers were synthesized by the Genomics Technology Platform (ESSBO, Oran, Algeria). This combined fingerprinting approach was used to compare the isolates at the strain level and to support the selection of non-redundant representatives for further analyses [39].

PCR amplifications followed the optimized protocol reported by [21]. Each 40 µL reaction contained approximately 100 ng of genomic DNA, 2× DreamTaq PCR Master Mix (Thermo Scientific, Waltham, MA, USA), DMSO, BSA, nuclease-free water, and the corresponding primers. For (GTG)_5_-PCR and BOX-PCR, 3 µL of 5 µM primer were used, whereas ERIC-PCRs contained 1.5 µL of each primer. Negative controls were included in each run, and amplification was performed for 35 cycles in a SimpliAmp thermal cycler (Applied Biosystems).

The PCR products were separated on 1.5% agarose gels prepared in 1× TBE buffer at 100 V for 2 h. Gels were stained with SYBR Safe and visualized using a ChemiDoc Imaging System (Bio-Rad, Hercules, CA, USA). GeneRuler Express and 1 kb Plus DNA ladders (Thermo Scientific, Waltham, MA, USA) were used as molecular size markers. To ensure reproducibility, each rep-PCR assay was repeated several times, and only reproducible banding patterns were retained for profile comparison and cluster analysis.

Banding profiles were analysed with GelJ software (version 2.0.0). Similarity matrices were generated using the Dice coefficient, and dendrograms were constructed by the UPGMA clustering method to assess genetic relationships among isolates [40,41].

### 2.3. PCR-Based Detection of Virulence-Associated, Antimicrobial Resistance, and Enterocin-Encoding Genes

To provide a preliminary molecular assessment of the safety-related and functional traits of the isolates, PCR-based screening was performed to detect selected virulence-associated genes, antibiotic resistance genes, and enterocin structural genes.

#### 2.3.1. Detection of Virulence-Associated and Antimicrobial Resistance Markers

All six isolates were first tested for five virulence-associated determinants: *cylM*, *cylA*, *cob*, *ccf*, and *gelE*. Isolates that were negative in this initial screening were then subjected to a second PCR screening targeting the *ddl* molecular marker, additional virulence-associated genes (*agg*, *hyl*, and *ace*) and selected antimicrobial resistance genes (*vanA*, *vanB*, *vanC1*, *ermA*, and *ermB*). Primer sequences, expected amplicon sizes, and annealing temperatures are provided in Table 1. As outlined in the Introduction, this targeted gene panel was selected to provide a preliminary PCR-based assessment of safety-related and functional traits in *Enterococcus* spp.

PCRs were carried out in a final volume of 25 µL containing 12.5 µL of 2× PCR Master Mix (Thermo Scientific, Waltham, MA, USA), 1 µL of each primer, 5 µL of genomic DNA corresponding to approximately 150 ng, and nuclease-free water. The cycling program started with denaturation at 94 °C for 5 min, followed by 35 cycles of 94 °C for 1 min, primer-specific annealing at the temperature indicated in Table 1 for 1 min, and extension at 72 °C for 1 min 45 s. A final extension was performed at 72 °C for 5 min. Amplicons were separated on 1.2% agarose gels. A GeneRuler 1 kb DNA Ladder (Thermo Scientific, Waltham, MA, USA) was used for size estimation. Gels were stained with SYBR Safe DNA Gel Stain (Thermo Scientific, Waltham, MA, USA) and visualized using a ChemiDoc Imaging System (Bio-Rad, Hercules, CA, USA). For the secondary screening of the two selected isolates, coded 9 and 13, 16S rRNA gene amplification was used as a DNA amplifiability control.

#### 2.3.2. Detection of Enterocin-Encoding Genes

Screening for enterocin structural genes (*entA*, *entB*, *entL50A*, *entL50B*, *ent31*, *entP*, *entAS-48*, and *entCRL35*) was carried out with the gene-specific primers listed in Table 1. The reaction mixture and electrophoretic analysis were the same as those described for virulence and antimicrobial resistance gene screening.

For enterocin genes, amplification was performed using a specific thermal program: 95 °C for 30 s, followed by 35 cycles of 95 °C for 30 s, primer-specific annealing at the temperature indicated in Table 1 for 30 s, and extension at 72 °C for 1 min 40 s. A final extension step was then performed at 72 °C for 7 min. For the secondary enterocin gene screening, amplification of the housekeeping gene *adk* was used as a DNA amplifiability control.

### 2.4. Antimicrobial Activity Assay

The antimicrobial activity of the two selected isolates, coded 9 and 13, which were negative for the targeted virulence-associated and antimicrobial resistance genes, was tested against nine ATCC indicator strains listed in Table 2. Antagonistic activity was assessed using an agar well diffusion assay adapted from [21].

The tested isolates were grown in MRS broth at 37 °C for 24 h. Indicator strains were cultured in Luria–Bertani broth (LB) (Merck, Darmstadt, Germany) under the same incubation conditions and adjusted to 0.5 McFarland turbidity, corresponding to approximately 1.5 × 10^8^ CFU/mL. These standardized suspensions were spread onto LB soft agar containing 0.75% agar.

Cell-free supernatants (CFS) were prepared from cultures of the two selected isolates, coded 9 and 13, by centrifugation at 10,000× *g* for 15 min at 4 °C. To limit inhibition related to acidification, particularly lactic acid produced during growth in MRS broth, the supernatants were adjusted to pH 7.0 with sterile 1 M NaOH. They were then sterilized by filtration through 0.22 µm syringe filters (Merck Millipore, Darmstadt, Germany).

Native supernatants (NS) corresponded to neutralized and filter-sterilized CFS used without further concentration. Concentrated supernatants (CS) were prepared by freeze-drying approximately 50 mL of neutralized and filter-sterilized CFS. The lyophilized material was then reconstituted in 1 mL of sterile MRS broth, resulting in an approximately fifty-fold concentration.

For the agar well diffusion assay, wells of 6 mm in diameter were filled with 100 µL of either NS or CS. Plates were kept at 4 °C for 2 h to allow diffusion, then incubated at 37 °C for 24 h. Inhibition was evaluated by measuring the diameter of the clear zones around the wells, including the 6 mm well diameter, and results were expressed in millimetres (mm). Sterile uninoculated MRS broth was used as the negative control.

To assess whether the inhibitory activity was associated with proteinaceous compounds, CFS were treated with trypsin at a final concentration of 1 mg/mL of cell-free supernatant (Sigma-Aldrich, Saint Louis, MO, USA). Treated samples were incubated at 37 °C for 2 h, after which trypsin was inactivated by heating at 100 °C for 3 min. Trypsin-treated and untreated samples were then tested using the same agar well diffusion assay against the same indicator strains [21].

### 2.5. Statistical Analysis

Experiments were carried out in triplicate, and data are presented as mean ± standard deviation (SD). Statistical comparisons were performed using one-way analysis of variance (ANOVA), followed by Tukey’s post hoc multiple comparison test. For antimicrobial activity assays, statistical comparisons were performed separately for each producing isolate and each indicator organism to evaluate the effect of supernatant concentration on inhibition zone diameters. Analyses were conducted with GraphPad Prism software (version 9.0.0; GraphPad Software, San Diego, CA, USA). Differences were considered statistically significant at *p* ≤ 0.05.

## 3. Results

### 3.1. Presumptive Identification of Enterococcal Isolates

The six camel milk isolates were first examined using basic phenotypic tests. All isolates were Gram-positive, catalase-negative cocci, in agreement with the general profile of lactic acid bacteria. Microscopic observation confirmed their coccoid morphology. The isolates grew in the presence of 4% and 6.5% NaCl, tolerated pH 9.6, and were able to grow at 45 °C. Together, these characteristics supported their presumptive assignment to the genus *Enterococcus* before molecular identification.

### 3.2. Confirmation of the Isolates as E. faecium

A combined proteomic and molecular identification strategy was applied to confirm the taxonomic status of the isolates.

#### 3.2.1. MALDI-TOF MS Identification

MALDI-TOF MS analysis identified all six isolates as *E. faecium*, with log(score) values ranging from 2.20 to 2.34. The individual log(score) values were 2.31, 2.26, 2.34, 2.21, 2.20, and 2.32 for the isolates coded 9, 10, 11, 12, 13, and 17, respectively. Since all scores were above the 2.0 threshold, the identifications were considered reliable at the species level. The Bacterial Test Standard (BTS) consistently produced scores above 2.0, confirming that the system was properly calibrated during the analyses.

#### 3.2.2. 16S rRNA-Based Confirmation

Partial 16S rRNA gene sequencing was used to support the MALDI-TOF MS identification. Sequence comparison confirmed that the six isolates belonged to *E. faecium*, in agreement with both the proteomic identification and the phenotypic profile. BLAST 2.17.0 analysis against the NCBI GenBank database showed 98–99% similarity with reference *E. faecium* sequences, based on approximately 700 bp of high-quality trimmed sequences.

### 3.3. Phylogenetic Relationships Among the Isolates

**Figure 1:**
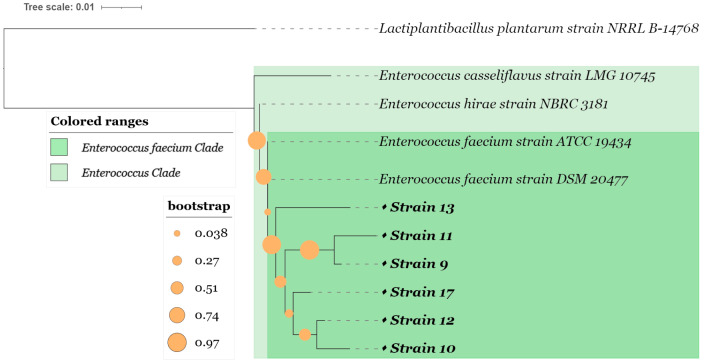
Phylogenetic tree of six *Enterococcus* isolates based on 16S rRNA sequences using the maximum likelihood method with 1000 bootstrap replicates, with *Lactiplantibacillus plantarum* as the outgroup.

Phylogenetic relationships among the six *Enterococcus* isolates recovered from Algerian camel milk were analysed using the Maximum Likelihood method based on partial 16S rRNA gene sequences. *Lactiplantibacillus plantarum* was used as the outgroup. The resulting tree separated the isolates within the *Enterococcus* genus and supported their taxonomic affiliation with *E. faecium* (Figure 1).

All six isolates, coded 9, 10, 11, 12, 13, and 17, clustered within the *E. faecium* group together with the reference strains *E. faecium* ATCC 19434 and DSM 20477. The short branch lengths observed among the isolates indicated close genetic relatedness, which is consistent with their classification within the same species. Slight differences in branch length may reflect limited intraspecific variation among the camel milk isolates.

The tree also distinguished *E. faecium* from the closely related species *Enterococcus casseliflavus* LMG 10745 and *Enterococcus hirae* NBRC 3181. These species formed separate neighbouring branches, while the outgroup, *Lactiplantibacillus plantarum*, was clearly positioned outside the *Enterococcus* cluster.

Overall, the phylogenetic analysis supported the identification of the camel milk isolates as *E. faecium* and complemented the MALDI-TOF MS and 16S rRNA sequence similarity results.

### 3.4. Genetic Diversity Revealed by rep-PCR

**Figure 2:**
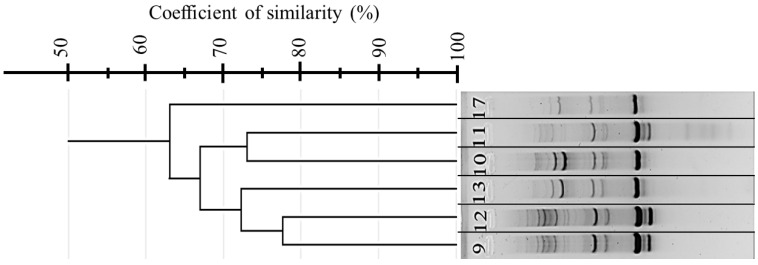
Dendrogram showing the genetic relatedness among six *E. faecium* isolates based on (GTG)_5_-PCR fingerprinting using the (GTG)_5_ primer. Clustering was performed using UPGMA and Dice similarity coefficients. Three distinct clusters were observed. Non-adjacent lanes from the same gel were spliced and reordered to match the dendrogram; splicing is indicated by visible separating lines.

**Figure 3:**
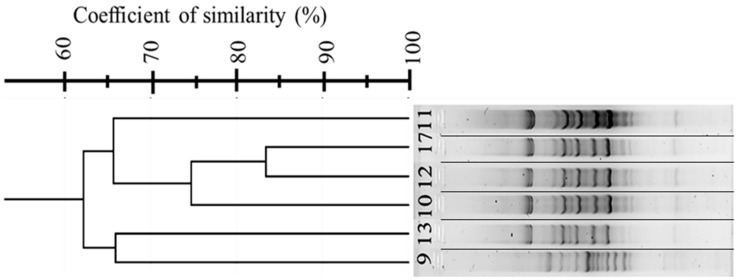
Dendrogram showing the genetic relatedness among six *E. faecium* isolates based on BOX-PCR fingerprinting using the BOXA1R primer. Clustering was performed with the UPGMA algorithm and Dice similarity coefficients. Resolution of three genetically distinct clusters was achieved.

**Figure 4:**
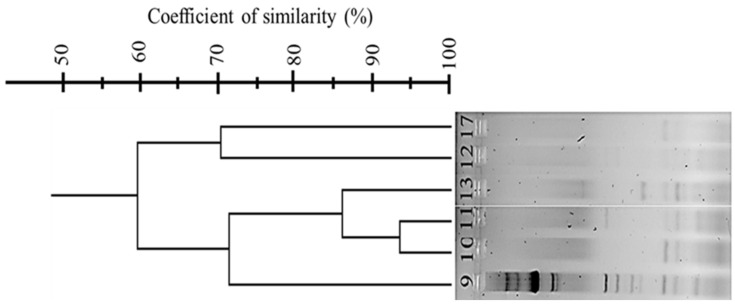
Dendrogram showing the genetic relatedness among six *E. faecium* isolates based on ERIC-PCR fingerprinting using primers ERIC1R and ERIC2. Clustering was performed using UPGMA and Dice similarity coefficients. Two major clusters were identified, indicating reduced discriminatory capacity compared to BOX-PCR and (GTG)_5_-PCR.

Genetic diversity among the six *E. faecium* isolates was investigated using three rep-PCR fingerprinting approaches: (GTG)_5_-PCR, BOX-PCR, and ERIC-PCR. The banding patterns were compared using the Dice similarity coefficient and clustered by the UPGMA method. The dendrograms obtained from the three approaches are shown in Figure 2, Figure 3 and Figure 4.

The (GTG)_5_-PCR profiles showed several distinct bands and allowed separation of the isolates into three clusters (Figure 2). Cluster A included isolates 9, 12, and 13, whereas isolates 10 and 11 were grouped in Cluster B. Isolate 17 formed a separate cluster, indicating a more distinct fingerprinting profile compared with the other isolates.

BOX-PCR produced more complex banding profiles and showed a higher discriminatory capacity than (GTG)_5_-PCR (Figure 3). Three clusters were obtained. Isolate 11 formed an independent cluster, while isolates 17, 12, and 10 clustered together. Isolates 13 and 9 formed a third cluster.

ERIC-PCR generated fewer bands than the two other rep-PCR methods and therefore showed lower resolution (Figure 4). This method separated the isolates into two clusters: one including isolates 9, 10, 11, and 13, and another including isolates 12 and 17. Despite its lower discriminatory power, ERIC-PCR showed partial agreement with the clustering patterns obtained using (GTG)_5_-PCR and BOX-PCR. Non-adjacent lanes from the same gel were spliced and reordered to match the dendrogram; splicing is indicated by visible separating lines.

### 3.5. Virulence-Associated and Antimicrobial Resistance Gene Profiles

**Figure 5:**
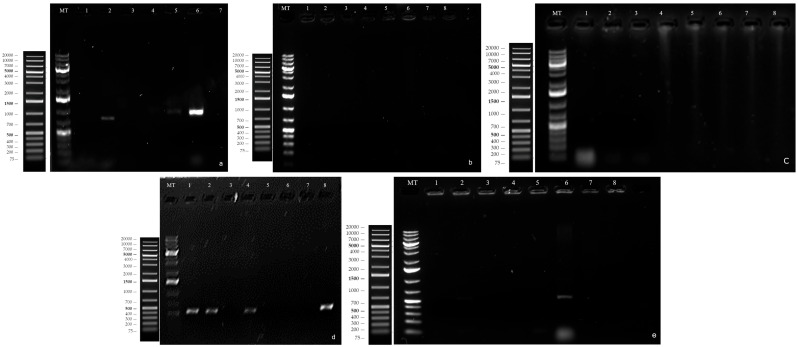
PCR screening of selected virulence-associated genes in *E. faecium* isolates. PCR products were resolved on 1.2% agarose gels. Panels: (**a**) *gelE*; (**b**) *cylM*; (**c**) *cylA*; (**d**) *ccf*; (**e**) *cob*. MT: DNA size marker, GeneRuler 1 kb DNA Ladder. Lane assignments: lane 1 and 2, controls; lane 3, isolate coded 9; lane 4, isolate coded 10; lane 5, isolate coded 11; lane 6, isolate coded 12; lane 7, isolate coded 13; lane 8, isolate coded 17. The isolates coded 9 and 13 were negative for all targeted virulence-associated genes.

PCR screening was used to evaluate selected safety-related molecular markers in the six *E. faecium* isolates. The first screening targeted five virulence-associated genes: *cylM*, *cylA*, *cob*, *ccf*, and *gelE* (Figure 5). The two isolates coded 9 and 13 were negative for all five targets. In contrast, the other isolates carried at least one of these genes: the isolate coded 10 was positive for *cob* and *ccf*, the isolate coded 11 for *gelE*, the isolate coded 12 for *cob* and *gelE*, and the isolate coded 17 for *ccf*.

**Figure 6:**
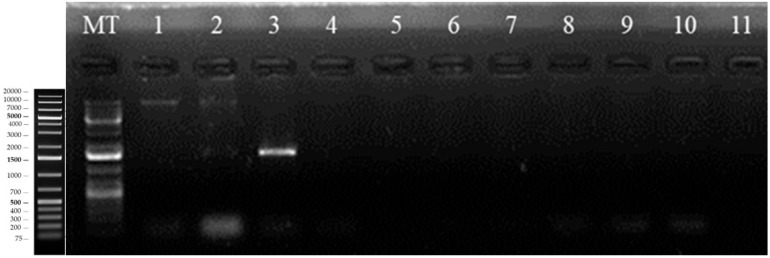
Secondary PCR screening of the *ddl* molecular marker and selected virulence-associated genes in the two selected isolates, coded 9 and 13. PCR products corresponding to *agg*, *ddl*, *hyl*, and *ace* were resolved on a 1.2% agarose gel. MT: DNA size marker, GeneRuler 1 kb DNA Ladder. Lane assignments: lane 1, genomic DNA from isolate coded 9; lane 2, genomic DNA from isolate coded 13; lane 3, 16S rRNA amplification control; lanes 4–5, *agg* screening for isolates coded 9 and 13; lanes 6–7, *ddl* screening for isolates coded 9 and 13; lanes 8–9, *hyl* screening for isolates coded 9 and 13; lanes 10–11, *ace* screening for isolates coded 9 and 13.

**Figure 7:**
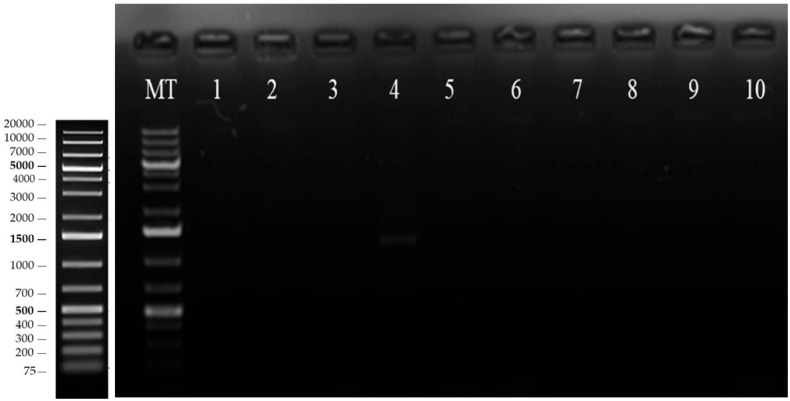
PCR screening of selected antimicrobial resistance genes in the two selected isolates, coded 9 and 13. PCR products corresponding to *vanA*, *vanB*, *vanC1*, *ermA*, and *ermB* were resolved on a 1.2% agarose gel. MT: DNA size marker, GeneRuler 1 kb DNA Ladder. Lane assignments: lanes 1–2, *vanA* screening for isolates coded 9 and 13; lanes 3–4, *vanB* screening for isolates coded 9 and 13; lanes 5–6, *vanC1* screening for isolates coded 9 and 13; lanes 7–8, *ermA* screening for isolates coded 9 and 13; lanes 9–10, *ermB* screening for isolates coded 9 and 13.

Because the isolates coded 9 and 13 lacked the initially screened virulence-associated genes, they were further analysed using an extended PCR panel. This second screening included the *ddl* molecular marker, additional selected virulence-associated genes (*agg*, *hyl*, and *ace*) (Figure 6), as well as selected antimicrobial resistance genes (*vanA*, *vanB*, *vanC1*, *ermA*, and *ermB*) (Figure 7). The 16S rRNA amplification control yielded the expected PCR product during this secondary screening. No PCR product was observed for the *ddl* molecular marker, the additional selected virulence-associated genes, or the selected antimicrobial resistance genes in either selected isolate. This PCR profile was therefore considered a preliminary molecular profile of interest and supported the selection of these two isolates for subsequent antimicrobial activity assays.

### 3.6. Enterocin Gene Profiles of the Selected Isolates

**Figure 8:**
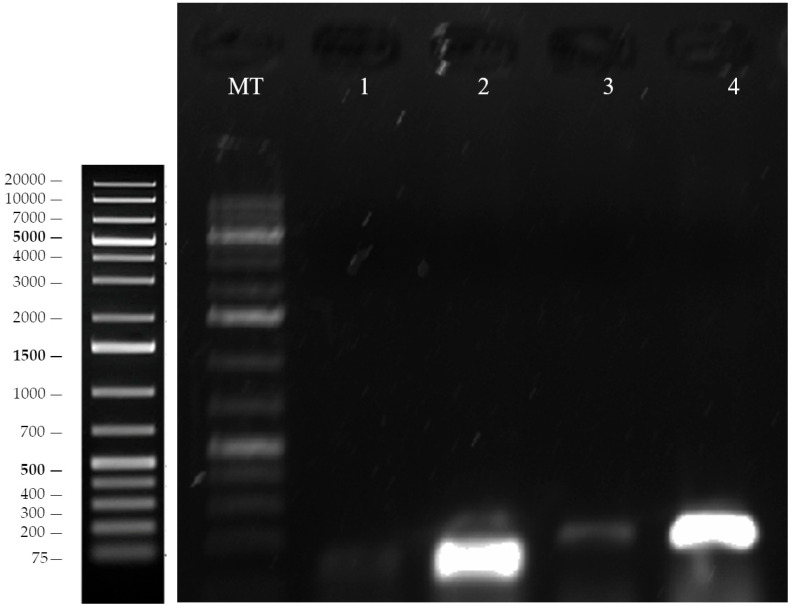
PCR screening of enterocin structural genes *entA* and *entB* in the two selected isolates, coded 9 and 13. PCR products were resolved on a 1.2% agarose gel. MT: DNA size marker, GeneRuler 1 kb DNA Ladder. Lane assignments: lanes 1–2, *entA* screening for isolates coded 9 and 13; lanes 3–4, *entB* screening for isolates coded 9 and 13.

**Figure 9:**
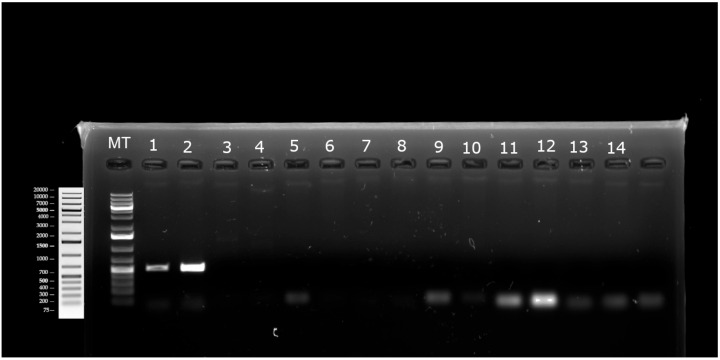
Secondary PCR screening of enterocin structural genes in the two selected isolates, coded 9 and 13. PCR products corresponding to *entL50A*, *entL50B*, *ent31*, *entP*, *entAS-48*, and *entCRL35* were resolved on a 1.2% agarose gel. MT: DNA size marker, GeneRuler 1 kb DNA Ladder. Lane assignments: lanes 1–2, *adk* amplification control for isolates coded 9 and 13; lanes 3–4, *entL50A* screening for isolates coded 9 and 13; lanes 5–6, *entL50B* screening for isolates coded 9 and 13; lanes 7–8, *ent31* screening for isolates coded 9 and 13; lanes 9–10, *entP* screening for isolates coded 9 and 13; lanes 11–12, *entAS-48* screening for isolates coded 9 and 13; lanes 13–14, *entCRL35* screening for isolates coded 9 and 13.

PCR screening was performed to detect enterocin structural genes in the tested isolates, including *entA*, *entB*, *entL50A*, *entL50B*, *ent31*, *entP*, *entAS-48*, and *entCRL35*. Among the screened isolates, only the two selected isolates coded 9 and 13 showed amplification for *entA* and *entB*, corresponding to enterocin A and enterocin B, respectively (Figure 8). No PCR product was observed for the other enterocin genes tested. The *adk* amplification control yielded the expected PCR product during the secondary enterocin gene screening (Figure 9). These results indicate that the two selected isolates carry genetic determinants associated with enterocin A and enterocin B.

### 3.7. Antimicrobial Activity of Native and Concentrated Supernatants

**Figure 10:**
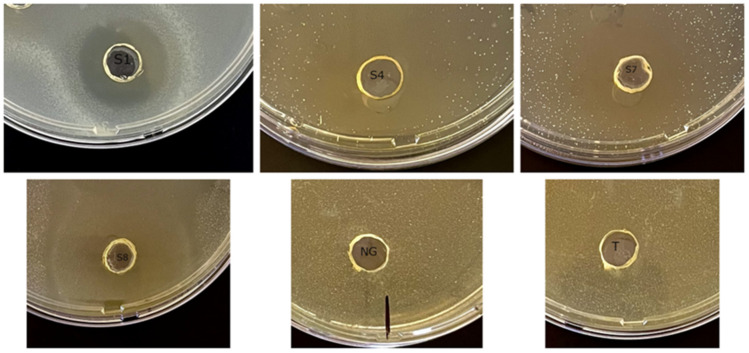
Antimicrobial activity of concentrated cell-free supernatant from the selected *E. faecium* isolate coded 9 against ATCC indicator strains. S1: methicillin-sensitive *Staphylococcus aureus* ATCC 29213; S4: *Selenomonas sputigena* ATCC 33150; S7: *Staphylococcus aureus* subsp. *aureus* ATCC 25923; S8: *Klebsiella pneumoniae* subsp. *pneumoniae* ATCC 13883. CS: concentrated supernatant; NG: negative control; T: trypsin-treated supernatant.

**Figure 11:**
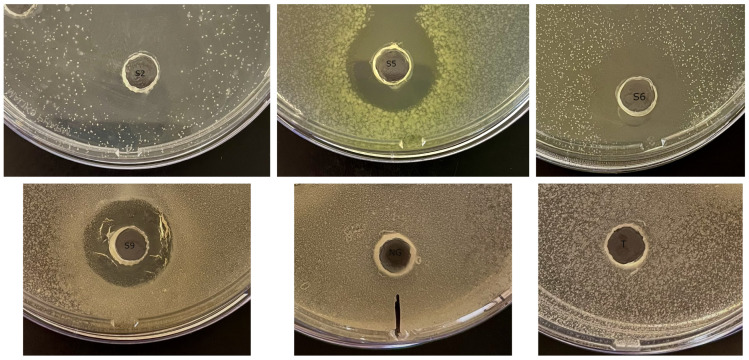
Antimicrobial activity of concentrated cell-free supernatant from the selected *E. faecium* isolate coded 13 against ATCC indicator strains. S2: *Salmonella enterica* subsp. *enterica* serovar *Typhimurium* ATCC 14028; S5: *Pseudomonas aeruginosa* ATCC 27853; S6: *Escherichia coli* ATCC 25922; S9: *Bacillus cereus* ATCC 11778. CS: concentrated supernatant; NG: negative control; T: trypsin-treated supernatant.

**Figure 12:**
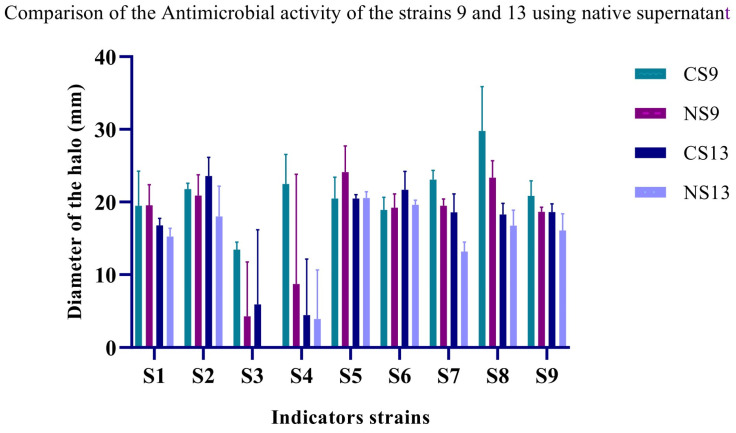
Comparative antimicrobial activity of native and concentrated cell-free supernatants from the two selected *E. faecium* isolates, coded 9 and 13, against ATCC indicator strains. NS: native supernatant; CS: concentrated supernatant; S1: methicillin-sensitive *Staphylococcus aureus* ATCC 29213; S2: *Salmonella enterica* subsp. *enterica* serovar Typhimurium ATCC 14028; S3: methicillin-resistant *Staphylococcus aureus* ATCC 43300; S4: *Selenomonas sputigena* ATCC 33150; S5: *Pseudomonas aeruginosa* ATCC 27853; S6: *Escherichia coli* ATCC 25922; S7: *Staphylococcus aureus* subsp. *aureus* ATCC 25923; S8: *Klebsiella pneumoniae* subsp. *pneumoniae* ATCC 13883; S9: *Bacillus cereus* ATCC 11778. Data represent mean inhibition zone diameters from triplicate assays.

The two isolates coded 9 and 13 were retained for antimicrobial activity testing because they were negative for the targeted virulence-associated and antimicrobial resistance genes. Their inhibitory activity was evaluated against nine ATCC indicator strains using the agar well diffusion assay (Figure 10, Figure 11 and Figure 12). In this assay, indicator strains showing clear inhibition zones were considered susceptible to the tested cell-free supernatants, whereas the absence of inhibition was interpreted as no detectable susceptibility under the assay conditions.

For the isolate coded 9, both native and concentrated cell-free supernatants inhibited the tested Gram-positive and Gram-negative indicator strains. Concentrated supernatants generally produced larger inhibition zones than native supernatants, with increases varying according to the indicator strain (Figure 10).

Among Gram-positive indicators, methicillin-resistant *Staphylococcus aureus* (MRSA, S3) showed weak and variable inhibition when tested with native supernatant. After concentration, inhibition increased markedly to 13.5 ± 1.0 mm. Increased inhibition after concentration was also observed for *Selenomonas sputigena* (S4).

*Klebsiella pneumoniae* subsp. *pneumoniae* (S8) was the most susceptible indicator strain, with inhibition zones of 23.4 ± 2.3 mm for native supernatant and 29.8 ± 6.1 mm for concentrated supernatant. The *Staphylococcus aureus* strains S1 and S7 were also clearly inhibited by the supernatants of the isolate coded 9.

Gram-negative indicators, including *Salmonella enterica*, *Escherichia coli*, and *Pseudomonas aeruginosa*, were inhibited by both native and concentrated supernatants, with larger zones generally observed after concentration. The isolate coded 13 also inhibited the tested indicator strains, but the inhibition zones were generally smaller and more variable than those observed with the isolate coded 9 (Figure 11). Concentration particularly enhanced the activity of the isolate coded 13 against *Salmonella enterica* (S2) and *S. aureus* (S7), whereas MRSA (S3) and *Selenomonas sputigena* (S4) showed weak or no detectable susceptibility to the native supernatant.

Trypsin treatment completely abolished the antimicrobial activity of both selected isolates, supporting the proteinaceous nature of the inhibitory compounds and indicating the possible involvement of bacteriocin-like substances. Statistical analysis using one-way ANOVA followed by Tukey’s post hoc test showed that concentration significantly increased the antimicrobial activity of the isolate coded 9 against MRSA (S3) (F(1,4) = 12.9, *p* = 0.022), *S. aureus* (S7) (F(1,4) = 21.3, *p* = 0.009), and *K. pneumoniae* (S8) (F(1,4) = 12.4, *p* = 0.023). For the isolate coded 13, a significant increase was observed only against *S. aureus* (S7) (F(1,4) = 7.8, *p* = 0.043), whereas the differences observed for the other indicator strains were not statistically significant.

Overall, the isolate coded 9 showed stronger and more consistent antimicrobial activity than the isolate coded 13, particularly after concentration of the supernatant. These findings support further investigation of both selected isolates as potential sources of proteinaceous antimicrobial compounds.

## 4. Discussion

This study provides a taxonomic and phylogenetic characterization of *E. faecium* isolates recovered from Algerian camel milk, a traditional dairy product known for its nutritional value and microbial diversity [42,43]. The isolates showed phenotypic traits compatible with the genus *Enterococcus*, including Gram-positive, catalase-negative coccoid morphology, tolerance to elevated NaCl concentrations, growth at alkaline pH, and ability to grow at 45 °C. These physiological characteristics are frequently reported among food-associated enterococci and may contribute to their adaptation and technological behaviour in dairy environments [44,45,46].

Species identification was supported by the combined use of MALDI-TOF MS and 16S rRNA gene sequencing. All isolates showed MALDI log(score) values ≥ 2.0, allowing species-level identification. Although partial 16S rRNA sequencing (~700 bp) has limited discriminatory power at the strain level because of the conserved nature of this gene, its agreement with the MALDI-TOF MS results supports the assignment of the isolates to *E. faecium* [18,47]. The minor sequence differences observed among isolates, together with the rep-PCR fingerprinting profiles described below, are consistent with intra-species diversity among the camel milk-derived *E. faecium* isolates [48,49]. Thus, the combination of proteomic and molecular identification provided complementary evidence for the taxonomic classification of the isolates.

Phylogenetic analysis based on the Maximum Likelihood method placed all camel milk-derived isolates within the *E. faecium* clade, in close association with the reference strains ATCC 19434 and DSM 20477. The short internal branch lengths suggest close genetic relatedness among the isolates, which is commonly observed among food-associated *E. faecium* populations recovered from dairy environments [50,51]. Their separation from neighbouring species, including *E. hirae* and *E. casseliflavus*, further supports the species-level assignment and is consistent with the taxonomic distinction observed within the genus *Enterococcus* [52,53].

Comparative genomic studies have shown that *E. faecium* includes distinct ecological lineages, particularly hospital-adapted populations often associated with antimicrobial resistance and virulence traits, and food-associated lineages generally considered to have lower pathogenic potential [54,55]. The clustering of the camel milk isolates is compatible with their origin from a food-associated environment. However, PCR-based screening remains a preliminary approach and cannot replace whole-genome sequencing (WGS). WGS would be required to define their genomic background more precisely and to assess the possible presence of mobile genetic elements or horizontally transferable resistance determinants, especially because *E. faecium* is known for its genomic plasticity [54]. Taken together, the taxonomic, phylogenetic, and molecular screening data support a preliminary classification of these isolates as food-associated *E. faecium* strains, while highlighting the need for genomic validation before any technological application.

Genotyping based on (GTG)_5_-PCR, BOX-PCR, and ERIC-PCR showed that the three fingerprinting methods differed in their discriminatory capacity. Both (GTG)_5_-PCR and BOX-PCR separated the isolates into three genetic clusters, with BOX-PCR providing the clearest resolution. This observation is in line with previous studies reporting the usefulness of BOX-PCR for distinguishing closely related food-derived *E. faecium* strains [56,57]. By contrast, ERIC-PCR produced fewer and less intense bands and separated the isolates into only two clusters. This lower resolution may be related to the distribution of ERIC sequences in the genome or to methodological sensitivity, as previously reported for enterococci [19,56]. The variability observed among the camel milk isolates is consistent with the intra-species heterogeneity described in both food and clinical *E. faecium* populations [58,59,60]. The use of three independent rep-PCR systems therefore provided complementary information and supported the presence of strain-level diversity within this dairy-associated group.

Virulence-associated determinants contribute to traits such as adhesion, biofilm formation, and host colonization, and are therefore important markers to consider when assessing the safety of food-associated *Enterococcus* strains [23,61,62]. In the present study, the two selected isolates, coded 9 and 13, were the only isolates negative for all selected virulence-associated genes (*cylM*, *cylA*, *agg*, *gelE*, *ace*, *hyl*, *cob*, and *ccf*), suggesting a lower burden of the investigated virulence-associated markers. These two selected isolates were also negative for the selected antimicrobial resistance genes (*vanA*, *vanB*, *vanC1*, *ermA*, and *ermB*), supporting a preliminary molecular profile of interest among the tested isolates.

This observation agrees with previous reports suggesting that food-associated *E. faecium* strains may present more favourable safety profiles than hospital-adapted lineages [19,63,64]. However, targeted PCR screening only covers a limited set of genes and cannot rule out the presence of additional resistance determinants, uncharacterized virulence factors, or mobile genetic elements. Therefore, whole-genome sequencing and phenotypic antimicrobial susceptibility testing would be necessary to complete the safety assessment of these isolates. The ability to produce biogenic amines is another relevant safety aspect for enterococci intended for food-related applications [65,66,67]. This parameter was not evaluated in the present work and should therefore be included in future investigations to provide a more complete safety profile.

PCR screening showed that the two selected isolates, coded 9 and 13, carried the enterocin structural genes *entA* and *entB*. Although amplification intensity differed between the two isolates, both showed detectable bands, indicating the presence of these bacteriocin-related genes. The detection of *entA* and *entB* suggests that these two selected isolates have the genetic potential to produce class II enterocins. Enterocins produced by *E. faecium*, including enterocins A and B, have been reported to act mainly against Gram-positive bacteria, particularly *Listeria* spp., *Enterococcus* spp., *Lactococcus* spp., and other lactic acid bacteria, although the activity spectrum may vary according to the producer strain and the target organism [68,69,70]. Nevertheless, the presence of enterocin genes alone does not demonstrate their expression or activity, since bacteriocin production may depend on regulation, culture conditions, and post-translational processing.

Both selected isolates inhibited Gram-positive and selected Gram-negative indicator organisms, with the isolate coded 9 showing the broadest and most consistent antimicrobial activity. Concentration of the cell-free supernatants increased the inhibitory effect, particularly against MRSA and *K. pneumoniae*. Since the supernatants were neutralized to pH 7.0 before testing, the inhibition observed is unlikely to be explained only by organic acid-mediated acidification. In addition, the complete loss of activity after trypsin treatment strongly supports the proteinaceous nature of the inhibitory compounds. Together with the clear inhibition zones observed against several indicator bacteria, these findings are consistent with the presence of bacteriocin-like antimicrobial substances in the active supernatants. The inhibition observed against Gram-negative bacteria is consistent with previous reports indicating that some enterocin-producing *E. faecium* strains may inhibit Gram-negative bacteria despite the outer membrane barrier. This activity may depend on bacteriocin concentration, the producer isolate, and the experimental conditions used [19].

The association of a preliminary molecular profile of interest with detectable antimicrobial activity supports the relevance of the two isolates, coded 9 and 13, for further investigation. Overall, the phenotypic, proteomic, molecular, genotyping, and antimicrobial data provide a coherent preliminary characterization of camel milk-derived *E. faecium* isolates. However, the study included a limited number of isolates, and the functional evaluation mainly focused on two selected isolates. Therefore, these findings should be interpreted with caution and should not be generalized to all camel milk-derived *E. faecium* populations.

The safety assessment was also based on targeted PCR screening of selected virulence-associated and antimicrobial resistance genes. Although the two selected isolates, coded 9 and 13, were negative for the markers investigated in this study, this does not exclude the presence of other resistance determinants, latent virulence factors, or mobile genetic elements not targeted by the PCR assays. Phenotypic antimicrobial susceptibility testing was not performed and should be included in future evaluations.

Further investigations are therefore required to better assess the biotechnological relevance and safety of these isolates. Whole-genome sequencing would provide a more complete view of their genomic background and would help evaluate genes or mobile elements associated with horizontal gene transfer, antimicrobial resistance, or virulence. Assessment of biogenic amine production should also be included, given its importance for food safety. From a functional point of view, purification and structural characterization of the antimicrobial compounds would help confirm their bacteriocin nature and clarify their activity spectrum. Additional mechanistic studies and validation in relevant food model systems would also be necessary before considering any practical food-related application.

Based on the current dataset, the two selected isolates, coded 9 and 13, should be considered camel milk-derived *E. faecium* isolates with antimicrobial potential that requires further genomic, safety, and functional investigation. Collectively, these results support the interest of Algerian camel milk as a source of *E. faecium* isolates with potential technological relevance and encourage further exploration of its microbial diversity for food biotechnology applications.

## 5. Conclusions

This study provides a preliminary integrated molecular and phenotypic characterization of camel milk-derived *E. faecium* isolates and highlights their antimicrobial potential. Among the six isolates investigated, the two selected isolates, coded 9 and 13, showed a preliminary molecular profile of interest, based on the absence of the selected virulence-associated and antimicrobial resistance markers investigated, together with detectable proteinaceous antimicrobial activity. Notably, the isolate coded 9 displayed a broader and more consistent antimicrobial activity than the isolate coded 13, despite carrying the same enterocin structural genes (*entA* and *entB*), which may reflect differences in gene expression, regulation, antimicrobial compound production, or other isolate-dependent factors. This finding emphasizes the importance of functional characterization in addition to gene detection.

Future work should focus on whole-genome sequencing, extended phenotypic safety assessment, assessment of biogenic amine production, characterization of the antimicrobial compounds, and validation in relevant food models to further support the technological evaluation of these isolates. Overall, these findings identify Algerian camel milk as a valuable source of *E. faecium* isolates with antimicrobial potential and support further investigation of their possible relevance for food biotechnology.

## Figures and Tables

**Table 1:** PCR primers and annealing temperatures used for molecular characterization of *Enterococcus* spp.

Gene	Role	Sequence Forward (5′–3′)	Sequence Reverse (5′–3′)	Product (bp *)	AT ** (°C)	References
MOLECULAR IDENTIFICATION						
16S rRNA	Genotypic identification	AGAGTTTGATCCTGGCTCAG	ACGGCTACCTTGTTACGACTT	1485	56	[20,21]
GENOTYPING (rep-PCR)						
ERIC	Repetitive element (ERIC-PCR)	ATGTAAGCTCCTGGGGATTCAC	AAGTAAGTGACTGGGGTGAGCG	Variable	52	[21]
(GTG)_5_	Repetitive element ((GTG)_5_-PCR)	GTGGTGGTGGTGGTG	–	Variable	45	[21]
BOXA1R	Repetitive element (BOX-PCR)	CTACGGCAAGGCGACGCTGACG	–	Variable	53	[21]
ADDITIONAL MOLECULAR MARKER						
*ddl*	D-Ala:D-Ala ligase marker	ATCAAGTACAGTTAGTCT	ACGATTCAAAGCTAACTG	941	50	[22]
VIRULENCE-ASSOCIATED GENES						
*agg*	Aggregation protein	AAGAAAAAGAAGTAGACCAAC	AAACGGCAAGACAAGTAAATA	1553	56	[23]
*hyl*	Hyaluronidase	ACAGAAGAGCTGCAGGAAATG	GACTGACGTCCAAGTTTCCAA	276	56	[24]
*gelE*	Gelatinase (metalloendopeptidase)	ACCCCGTATCATTGGTTT	ACGCATTGCTTTTCCATC	419	54	[25,26]
*cylM*	Cytolysin modification protein	CTGATGGAAAGAAGATAGTAT	TGAGTTGGTCTGATTACATTT	742	54	[23]
*cylA*	Cytolysin activation protein	ACTCGGGGATTGATAGGC	GCTGCTAAAGCTGCGCTT	688	54	[27]
*ace*	Collagen-binding protein	GAATGACCGAGAACGATGGC	CTTGATGTTGGCCTGCTTCC	615	58	[28]
*cob*	Sex pheromone	AACATTCAGCAAACAAAGC	TTGTCATAAAGAGTGGTCAT	1405	54	[23]
*ccf*	Sex pheromone	GGGAATTGAGTAGTGAAGAAG	AGCCGCTAAAATCGGTAAAAT	543	54	[23,29]
ANTIBIOTIC RESISTANCE GENES						
*vanA*	Vancomycin resistance (D-Ala–D-Lac ligase)	CATGAATAGAATAAAAGTTGCAATA	CCCCTTTAACGCTAATACGATCAA	1030	54	[19,30]
*vanB*	Vancomycin resistance	GTGACAAACCGGAGGCGAGGA	CCGCCATCCTCCTGGAAAAAA	433	54	[19,30]
*vanC1*	Vancomycin resistance	GGTATCAAGGAAACCTC	CTTCCGCCATGGCAGTAT	822	54	[30]
*ermA*	Erythromycin resistance (rRNA methylase)	TCTAAAAAGCATGTAAAAGAA	TGATTATAATTATTTGATAGCTTC	645	52	[19,31]
*ermB*	Erythromycin resistance (rRNA methylase)	GAAAAGGTACTCAACCAAATA	CATTTGTTAAATTCATGGCAATGA	639	52	[31,32]
ENTEROCIN STRUCTURAL GENES						
*entA*	Enterocin A	GGTACCACTCATAGTGGAAA	CCCTGGAATTGCTCCACCTAA	138	55	[19,33]
*entB*	Enterocin B	CAAAATGTAAAAGAATTAAGTACG	AGAGTATACATTTGCTAACCC	201	55	[34]
*entL50A*	Enterocin L50A	ATGGGAGCAATCGCAAAATTA	TTTGTTAATTGCCCATCCTTC	135	56	[5,19]
*entL50B*	Enterocin L50B	ATGGGAGCAATCGCAAAATTA	TAGCCATTTTTCAATTTGATC	274	58	[33]
*entP*	Enterocin P	ATGAGAAAAAAATTATTTAGTTT	TTAATGTCCCATACCTGCCAAACCAG	216	41	[19]
*entAS-48*	Enterocin AS-48	GAGGAGTATCATGGTTAAAGA	ATATTGTTAAATTACCAA	339	56	[19,35]
*ent31*	Enterocin 31	CCTACGTATTACGGAAATGGT	GCCATGTTGTACCCAACCATT	130	58	[19]
*entCRL35*	Enterocin CRL35	GCAAACCGATAAGAATGTGGGAT	TATACATTGTCCCCACAACC	490	55	[19]

*: base pairs; **: annealing temperature.

**Table 2:** Indicator strains used for antimicrobial activity evaluation.

Strain Code	Designation
S1	*Staphylococcus aureus* ATCC 29213 (methicillin-sensitive)
S2	*Salmonella enterica* subsp. *enterica* serovar *Typhimurium* ATCC 14028
S3	*Staphylococcus aureus* (methicillin-resistant) ATCC 43300
S4	*Selenomonas sputigena* ATCC 33150
S5	*Pseudomonas aeruginosa* ATCC 27853
S6	*Escherichia coli* ATCC 25922
S7	*Staphylococcus aureus* subsp. *aureus* ATCC 25923
S8	*Klebsiella pneumoniae* subsp. *pneumoniae* ATCC 13883
S9	*Bacillus cereus* ATCC 11778

## Data Availability

The datasets generated and/or analyzed during the current study, including the partial 16S rRNA gene sequences, are available from the correspondence author upon reasonable request.
